# Surgical injury and repair of hip external rotators in THA via posterior approach: a three-dimensional MRI-evident quantitative prospective study

**DOI:** 10.1186/s12891-018-2367-1

**Published:** 2019-01-14

**Authors:** Ting Wang, Long Shao, Wei Xu, Feilong Li, Wei Huang

**Affiliations:** Department of Orthopedics, The First Affiliated Hospital of Chongqing Medical University, No.1 Youyi Road, Yuan jiagang, Yuzhong District, Chongqing, China

**Keywords:** Total hip arthroplasty, External rotators, Three-dimensional MRI reconstruction, Injury, Repair

## Abstract

**Background:**

As one of the classical total hip arthroplasty (THA) approaches, the posterior approach is widely used. However, there is a lack of in-depth quantitative researches on the surgical-related injury to the hip external rotators. The purpose of this study is to quantificationally analyse the surgical injury of hip external rotators after posterior THA and explore the effect of the muscle repair on the muscle recovery using the MRI three-dimensional reconstruction technique combined with the clinical assessment.

**Methods:**

Sixty five patients were eligible to receive a unilateral cementless THA via the posterior approach. During operation, the piriformis tendon was reattached but it was not applicable for the internal obturator muscle. We performed three-dimensional MRI reconstruction of bilateral piriformis and internal obturator muscle along with clinical assessment preoperatively, 6, 12 and 52 weeks postoperatively.

**Results:**

Bilateral piriformis and internal obturator muscle were homogeneous preoperatively. Compared with the contralateral side, the volume atrophy and fat-muscle ratio of the piriformis on the operated side increased inconspicuously by 1.64%, 0.26% (*p* = 0.062, *p* = 0.071) at 6 weeks and 1.33%, 0.20% (*p* = 0.057, *p* = 0.058) at 12 weeks, while 7.28%, 2.09% and 15.71%, 5.14% for the internal obturator muscle (*p* < 0.01). Up to 52 weeks, the pirformis also showed significant muscle atrophy as well as fatty infiltration (increased by 7.79%, 4.21%; *p* < 0.01), and 24.18%, 11.91% for the internal obturator muscle (*p* < 0.01).

**Conclusion:**

The THA via posterior approach significantly harms the hip external rotators and the early hip external rotation function. The effective repair could be conducive to the early postoperative recovery of the hip external rotators.

**Trail registration:**

The study has been registered in Chinese Clinical Trial Registry (ChiCTR) before the clical trial started, the Clinical Trial Registry Number is ChiCTR-IOR-17013007. Registered 17 October 2017. The Trial registration is prospective registration.

## Background

Total hip arthroplasty (THA) is one of the most successful orthopedic surgery in the twentieth century; it is the preferred treatment for end-stage coxarthropathy. The posterior approach, also known as the “Kocher-Langenbeck approach”, is the most frequently used among multitudinous THA approaches [1, 2]. Concerning this approach, a widespread controversy has been always existed over the surgical injury of the hip external rotators and whether to repair them or not.

Various studies have confirmed the muscle damage to the short external rotators via posterior THA from cadaveric tests, electromyography (EMG), biochemical serum markers and gait analysis [3–6]. However, there was still a lack of consensus on the extent of the muscle damage and the necessity of the muscle repair. With the development of imaging technology, magnetic resonance imaging (MRI) has been the gold standard for muscles evaluation, especially the muscular morphorlogical changes [7, 8]. Nevertheless, to the authors’ knowledge, that of the hip external rotators was barely mentioned. In addition, all of these studies were based on the two-dimensional MRI single layer analysis so that the results lacked precision and objectiveness [9].

Given all this, the aim of this study was to address the following questions using the MRI three-dimensional reconstruction technique in combination with the clinical assessment: 1) What were the changes in the muscle morphology of hip external rotators and the external rotation function after posterior THA? 2) Whether the repair of external rotators contributed to a better amelioration in the muscle morphology postoperatively?

## Methods

### Patients

**Fig. 1:**
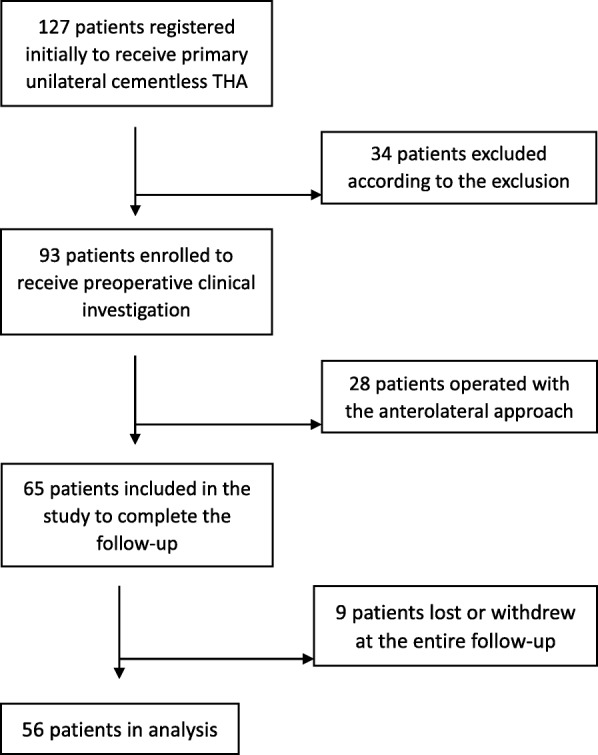
Flow chart showing screening of patients

This prospective study has been approved by Institutional Review Board and registered in Chinese Clinical Trial Registry (ChiCTR) before the clical trial started. The methods were carried out in accordance with the relevant guidelines and regulations. In this study, 127 patients prepared for unilateral THA were enrolled continuously from January 2018 to June 2018 including 74 males and 53 females. Each participant has signed the informed consent, and all were over 16 years old. Inclusion criteria comprises admission diagnosis for the development of hip dysplasia (Crowe type I, type II), avascular necrosis of the femoral head, primary hip osteoarthritis, femoral head epiphyseal Ischemic necrosis (Legg-Calve-Perthes disease). Exclusion criteria consists of contralateral hip disease or surgical history, joint ankylosis or stiffness, severe developmental hip dysplasia (Crowe type III, type IV), femoral neck fracture, intertrochanteric fractures, suppurative coxarthritis, rheumatoid arthritis, femoral or acetabular osteotomy, ipsilateral surgical history, severe systemic infection or tumor diseases, severe medical diseases, muscle weakness, muscle dystrophy or muscle atrophy related diseases, physical disability or mental illness. At last, 56 patients were eligible for the final analysis in this study (Fig. 1). Demographic data were registered (Table 1).

**Table 1:** Demographic Data

Demographic	
No. of patients	56
Age(yrs)	56.77 ± 15.44
Gender	
Male	33 (58.92%)
Female	23 (41.08%)
Height(cm)	164.05 ± 6.47
Weight(kg)	65.66 ± 10.08
BMI(kg/m^2^)	24.4 ± 3.8
Surgical side	
Left	32 (57.14%)
Right	24 (42.86%)
Operative time(min)	89.88 ± 14.75
Blood loss(ml)	145.54 ± 66.93
Incision length(cm)	9.16 ± 0.83
Diagnosis	
ANFH	29 (51.79%)
DDH	22 (39.28%)
OA	3 (5.35%)
Legg-Calve-Perthes	2 (3.58%)

*BMI* Body Mass Index, *ANFH* Avascular Necrosis of Femoral Head, *DDH* Developmental Dysplasia of Hip, *OA* Osteoarthritis

### Surgical intervention

**Fig. 2:**
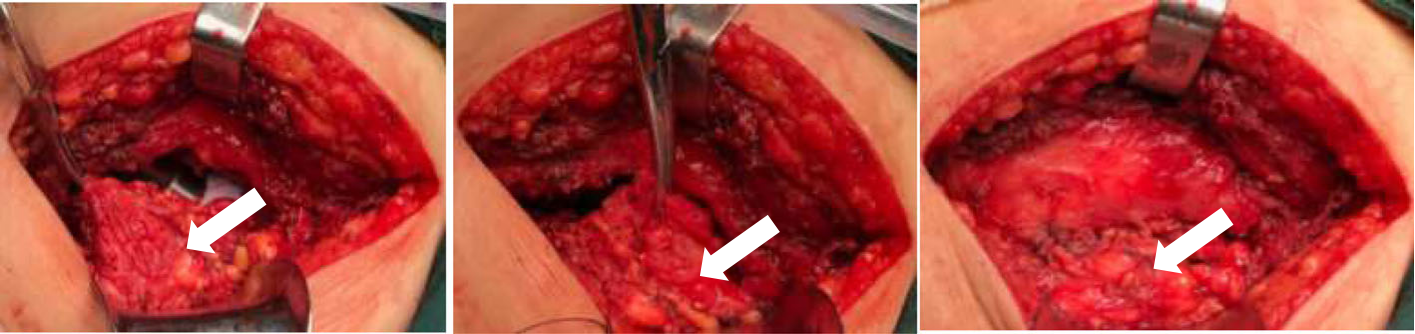
**a** The flap-shaped incision of posterior joint capsule. **b** The detachment of piriformis tendon. **c** The non-in-situ reattachment of piriformis in combination with the posterior joint capsule on the posterior part of femoral great trochanter

Each posterior THA was performed by the same senior medical practitioner. The prosthesis for each patient were all biological type. The material of femoral stem (LCU or Ribbed) and acetabular cup (T.O.P or Combi cup) were titanium alloy. In addition, the acetabular lining was high-crosslinked polyethylene and the femoral head was ceramic. During operation, the tendon insertion of the short external rotators group including piriformis, internal obturator muscle, superior gemellus, and inferior gemellus were cut off; the posterior joint capsule was cut through with a flap-shaped incision. Only the tendon of the piriformis in combination with the posterior joint capsule was non-in-situ reattached through a suture hole on the posterior part of femoral great trochanter using the non-absorbable suture (Ethibond) (Fig. 2). The same postoperative rehabilitation protocol was followed by each patient.

### Three-dimensional MRI reconstruction technique

**Fig. 3:**
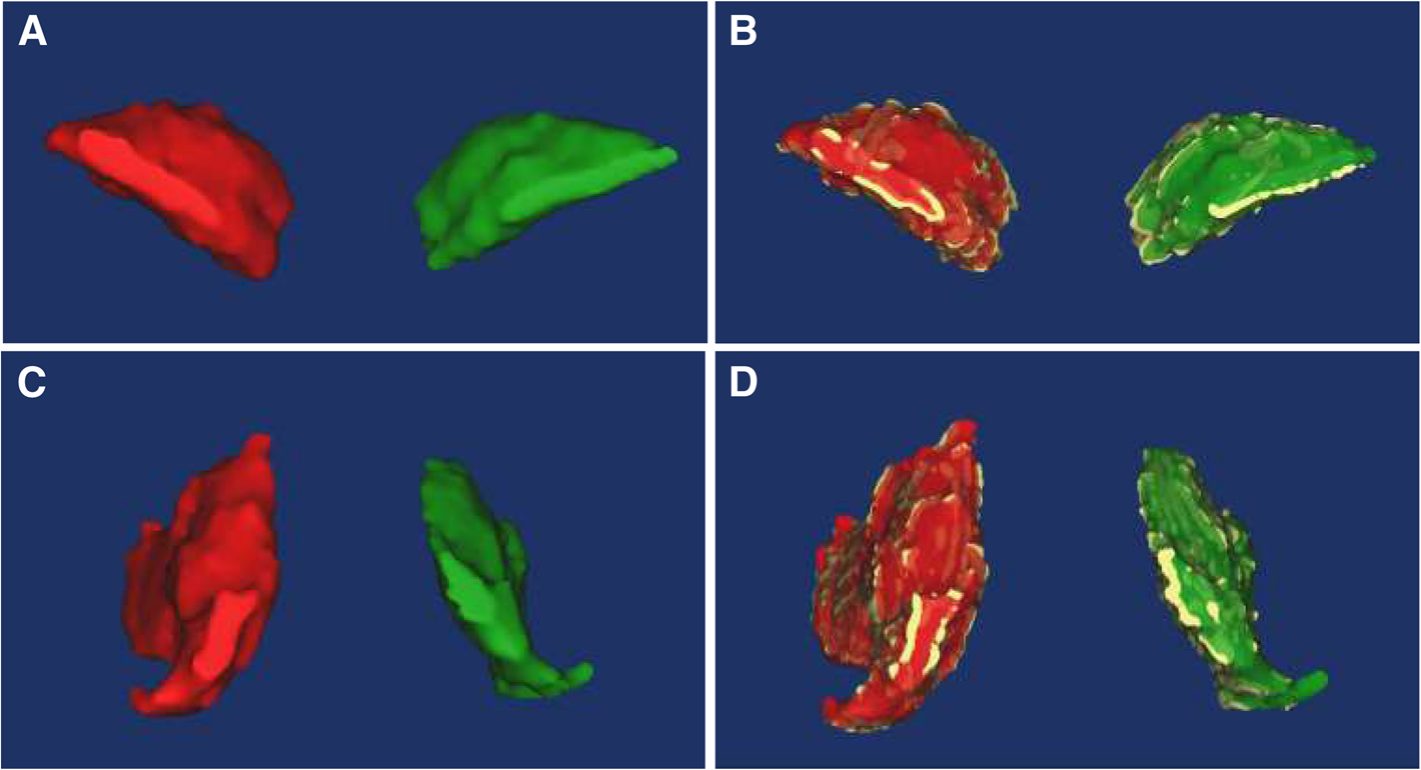
**a** The typical three-dimensional MRI reconstruction of bilateral piriformis. **b** The typical three-dimensional MRI reconstruction of bilateral piriformis with adipose tissue. **c** The typical three-dimensional MRI reconstruction of bilateral internal obturator muscle. **d** The typical three-dimensional MRI reconstruction of bilateral internal obturator muscle with adipose tissue. (The yellow represents adipose tissue)

MRI was performed on a 1.5 T scanner (Signa HDxt, GE MEDICAL SYSTEMS, USA) according to a standard protocol preoperatively, 6, 12 and 52 weeks postoperatively. Images were acquired in 4-mm slices using a flexible phased-array coil and clinically established MRI sequences at radiology department. T1-weighted fast spin-echo (FSE) images were obtained using configured scan parameters (TE = Min Full; TR = 760 ms; matrix size =512*512; bandwidth = 31.25 kHz; field of view = 480 mm; slice thickness = 3 mm; spacing = 1 mm). T2-weighted fast recovery with fast spin-echo (FRFSE) sequence was also acquired (Auto TR, 2820 ms; Echo Train Length (18 mm); matrix size, 512*512; bandwidth, 31.25 kHz; field of view, 480 mm; Freq, 228 kHz; slice thickness, 0.5 mm; spacing, 1 mm). The use of cross sections established the first cut from the level of anterior superior iliac spine to the middle of femoral shaft in each patient. The DICOM (Digital Imaging and Communications in Medicine) data of all the 56 bilateral hip joint MRI images were saved as DVD and loaded into Mimics 17.0 (Materialise, Belgium). Two researchers independently identified the contours of the bilateral external rotators using the LiveWare tool. If controversy existed over the identification between two researchers, the third professional veteran musculoskeletal radiologist with ten years of experience would make the final determination. The range of the reconstruction included all sections of the target muscle that can be clearly recognized on the cross-section. Due to the influence of muscle atrophy, the number of bilateral sections can vary. The Cronbach’s Alpha and intraclass correlation coefficient for consistency between the two judgers on each thresholding value selection for all included MRI images was 0.992 and 0.983 (95% CI = 0.972–0.990, F = 120.026, *p* = 0.000), respectively. After selection of adipose thresholding, “negative value” operation was applied in the course of the adipose tissue reconstruction in order to ensure that the range of the two masks was exactly the same. The adipose tissue was separated from the muscle through the Boolean operation. The volume of muscle, adipose tissue and the corresponding fat-muscle ratio were calculated from the polygonal surfaces on the basis of the three-dimensional reconstruction technique (Fig. 3).

### Clinical assessment

Bilateral hip external rotation range measurement was conducted on each patient by the same medical practitioner preoperatively, 6, 12 and 52 weeks postoperatively. The medical practitioner was blinded to the surgical procedure and the MRI results.

### Statistical analysis

Data analysis was performed by an independent statistician using SPSS (Version 22; SPSS Inc., Chicago, IL). Univariate analysis of continuous variables were compared with a parametric paired t test or a nonparametric Mann-Whitney U test. Because of the relatively small sample size, continuous variables were tested on normality using a Shapiro–Wilk test. When the *p* value was less than 0.05, data were assumed to not be normally distributed, and the nonparametric test was used. Since the study was self-paired design, the formula “n = [(Z_α/2_ + Z_β_)S/δ]^2^” was used for the calculation of the sample size (α = 0.05, β = 0.1). A p value less than 0.05 was deemed to be statistic significant.

## Results

### External rotators morphology analysis by three-dimensional MRI reconstruction

#### External rotators volume

**Fig. 4:**
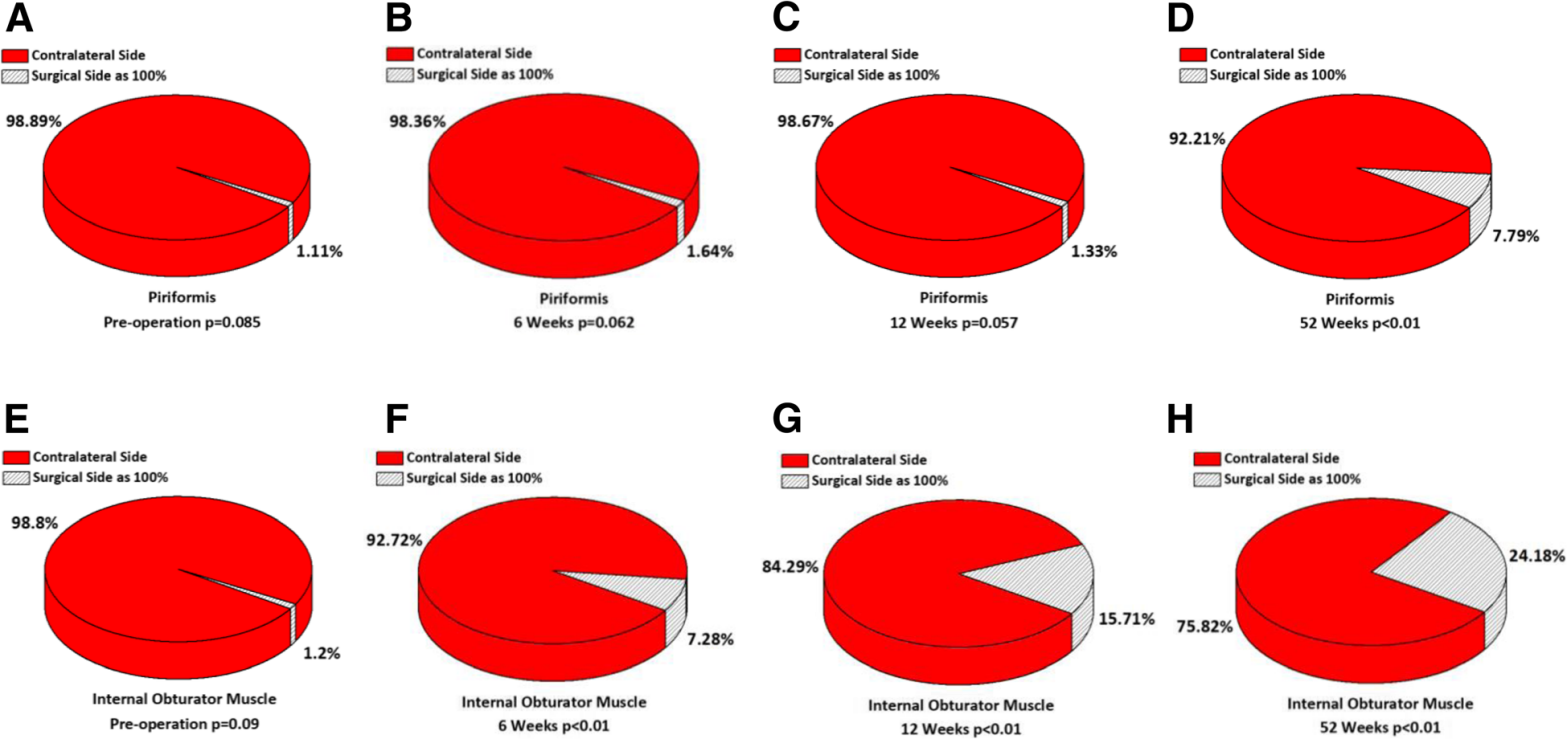
The percentage of muscles volume on the operated side in comparison with the contralateral side from pre-operation to 52 weeks postoperatively. **a**-**d** The muscle volume of piriformis decreased by 1.11% (*p* = 0.085) preoperatively, 1.64% (*p* = 0.062) at 6 weeks, 1.33% (*p* = 0.057) at 12 weeks and 7.79% (*p* < 0.01) at 52 weeks. **e-h** The muscle volume of internal obturator muscle decreased by 1.20% (*p* = 0.09) preoperatively, 7.28% (*p* < 0.01) at 6 weeks, 15.71% (*p* < 0.01) at 12 weeks and 24.18% (*p* < 0.01) at 52 weeks

There was not a significant difference regarding the muscle volume of the bilateral piriformis and internal obturator muscle preoperatively (piriformis: *p* = 0.085; internal obturator muscle: *p* = 0.09). At 6 weeks postoperatively, compared with the contralateral side, the muscle volume of piriformis on the operated side decreased by 1.64% (*p* = 0.062), while 7.28% for the internal obturator muscle (*p* < 0.01). At 12 weeks, the piriformis atrophy was still inapparent (decreased by 1.33%, *p* = 0.057); nevertheless, the muscle volume of internal obturator muscle continued to reduce (decreased by 15.71%, *p* < 0.01). Up to 52 weeks, the pirformis also showed significant muscle atrophy, which decreased by 7.79% (*p* < 0.01), and 24.18% for internal obturator muscle (*p* < 0.01) (Fig. 4). There was no significant change in the muscle volume on the contralateral side during the entire follow-up (*p* > 0.05).

#### External rotators fat-muscle ratio

**Fig. 5:**
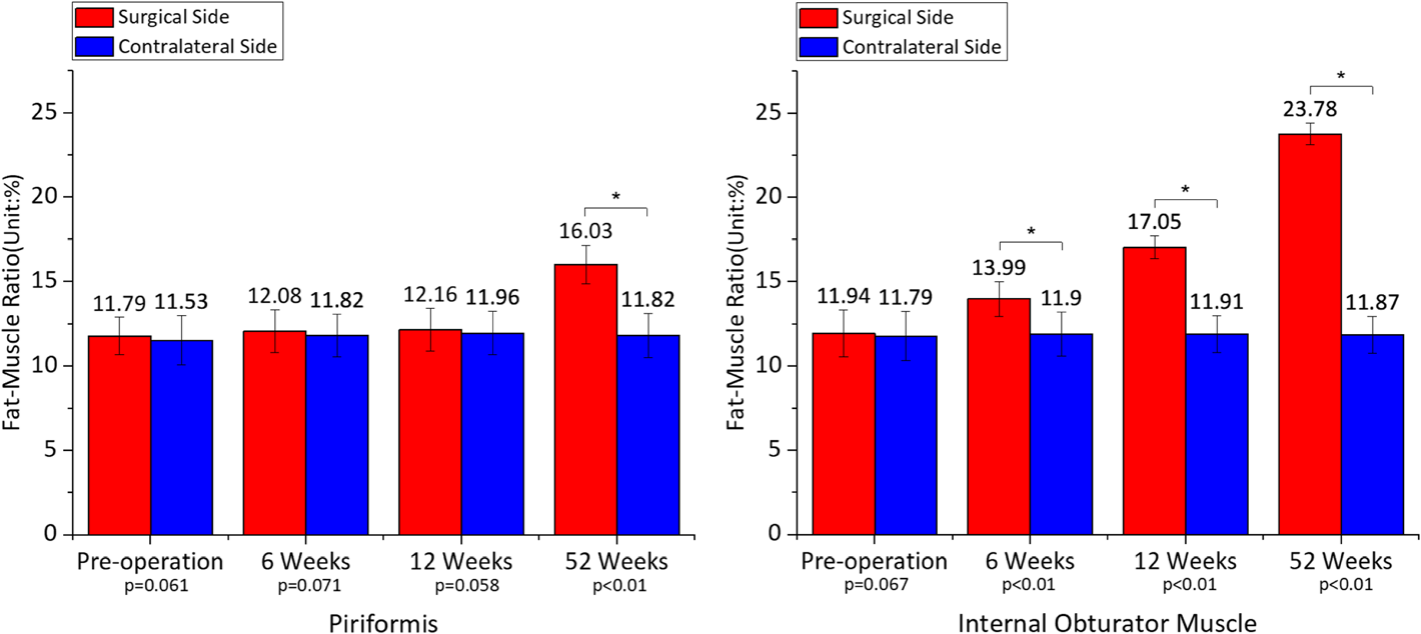
Mean ± standard deviation of fat-muscle ratio on the operated side in comparison with the contralateral side from pre-operation to 52 weeks postoperatively. **a** The fat-muscle ratio of piriformis increased by 0.26% (*p* = 0.061) preoperatively, 0.26% (*p* = 0.071) at 6 weeks, 0.20% (*p* = 0.058) at 12 weeks and 4.21% (*p* < 0.01) at 52 weeks. **b** The fat-muscle ratio of internal obturator muscle increased by 0.15% (*p* = 0.067) preoperatively, 2.09% (*p* < 0.01) at 6 weeks, 5.14% (*p* < 0.01) at 12 weeks and 11.91% (*p* < 0.01) at 52 weeks

No statistical significance (piriformis: *p* = 0.061; internal obturator muscle: *p* = 0.067) was found in the fat-muscle ratio of bilateral external rotators preoperatively. At 6 weeks postoperatively, the fat-muscle ratio of piriformis on the operated side only increased by 0.26% (*p* = 0.071) in comparison with the contralateral side; however, the ratio of internal obturator muscle went up markedly (increased by 2.09%, *p* < 0.01). At 12 weeks, the fatty infiltration degree of piriformis remained stable, which increased by 0.20%; and the ever-increasing ratio of internal obturator muscle presented more significant (increased by 5.14%, *p* < 0.01). At 52 weeks, both the piriformis and the internal obturator muscle manifested the dramatic augmentation in the fat-muscle ratio (piriformis: 4.21%, *p* < 0.01; internal obturator muscle: 11.91%, *p* < 0.01) (Fig. 5). There was no obvious change in the fat-muscle ratio on the contralateral side during the entire follow-up (*p* > 0.05).

### Clinical assessment

#### External rotation range

**Fig. 6:**
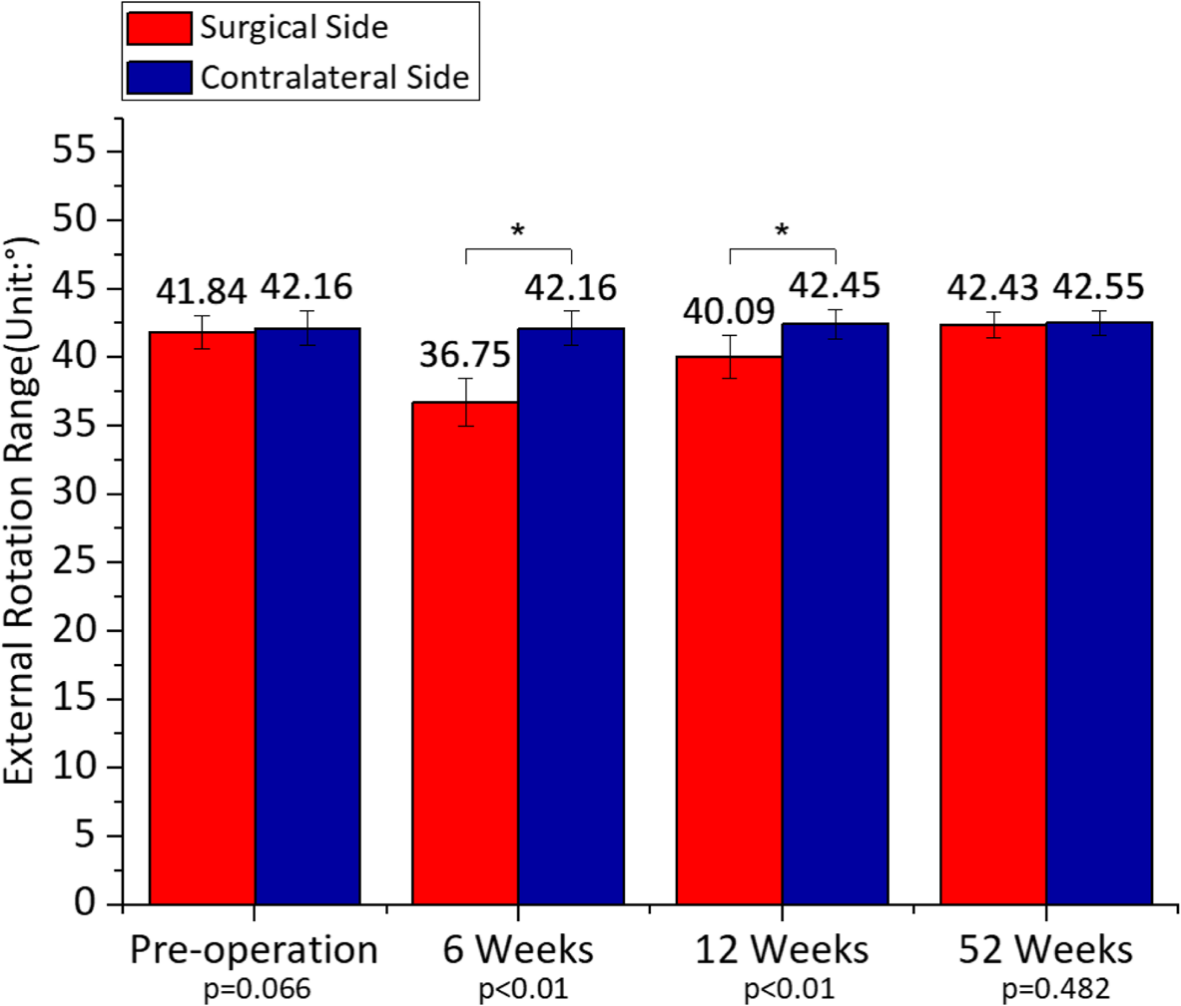
Mean ± standard deviation of external rotation range on the operated side in comparison with the contralateral side from pre-operation to 52 weeks postoperatively. The external rotation range decreased by 0.76% (*p* = 0.066) preoperatively, 12.84% (*p* < 0.01) at 6 weeks, 5.56% (*p* < 0.01) at 12 weeks and 0.29% (*p* = 0.482) at 52 weeks

The external rotation range of the bilateral hip joint was at the same baseline level preoperatively (*p* = 0.066), while it was limited obviously on the operated side at 6 weeks postoperatively (average of 36.75°, *p* < 0.01). At 12 weeks, it got closed to the normal value, but the difference was still significant (average of 40.09°, *p* < 0.01). After 52 weeks of rehabilitation, the external rotation angle completely recovered (average of 42.43°, *p* = 0.482) (Fig. 6). There was no statistical significance in the hip external rotation range on the contralateral side during the entire follow-up (*p* > 0.05).

## Discussion

The posterior approach has been used most frequently among various THA approaches [10, 11]; it holds the advantages of clear anatomical layer, intact hip abductors, lower incidence of heterotopic ossification, and short learning cycle over the others. However, controversy still exists over the degree of approach-related injury to the hip external rotators as well as whether to repair them or not. The results of this study proved more precisely that the posterior approach did seriously damage to the muscle morphology of hip external rotators as well as the external rotation function. Moreover, the effective muscle repair was beneficial to the amelioration of the muscular morphological insufficiency.

Previous researches have already confirmed surgical injury to the external rotators via posterior THA [12–16]. Several pathologic researches have already identified that loss of tendon-bone adhesion could lead to muscle atrophy and fatty infiltration [17, 18]. In theory, there was no doubt that the detachment of the muscle tendon intraoperatively could cause the injury to the short external rotators. Still, the research conclusions without muscle morphology analysis could not be entirely convincing. MRI is currently the gold standard for analyzing muscle damage through morphological changes, mainly including the muscle volume atrophy and fatty infiltration [19–21]. Rarely, Khan RJ et al. reported significant deterioration in the muscle grade and nearly 50% decrease in the muscle bulk of the piriformis via posterior approach through the two-dimensional MRI measurements [22]. Nevertheless, since two-dimensional measurement was limited to one or some specified MRI slices, a great loss of the key information of muscles was inevitable and then the results lacked accuracy and repeatability. Vidt ME et al. demonstrated that assessments of fatty infiltration and muscle atrophy from a single magnetic resonance image slice could not be predictive of 3-dimensional measurements [9]. In order to cover the shortage, our study took advantage of both accurate MRI morphology analysis and computer-assisted 3D reconstruction technique. Therefore, this study not only confirmed the previous research conclusions, but also further presented the extent of the muscle injury quantificationally. According to the authors’ knowledge, the general idea and realization of the digital 3D-reconstruction of skeletal muscles based on MRI raw data is reported for the first time.

Muscle injury is inevitable, so how to minimize the injury seems more important. The previous studies related to the surgical injury of external rotators, whether to repair the muscles or not and the effect of repair provoked some other controversy. Researchers in favor of the repair claimed that it was necessary because the repaired muscles provided a physical scaffold-like support to stabilize the hip joint and decrease the early postoperative prosthetic dislocation rate [23–26]. Meanwhile, the more balanced hip joint motion as well as the better external rotation function were also mentioned [27]. Nevertheless, with a great risk for re-rupture and failure, the opponents against the posterior structure repair stood by the theory that the repaired posterior structure could not meet the strength of daily routine activity and some actually insisted that the repair itself was not responsible for the lower dislocation rate [28–30]. Undoubtedly, the morphology and the quality of the short external rotators directly determine the muscular function as well as the joint stability [31]. Nevertheless, few studies have focused on its changes whether did the repair or not. Consequently, this study quantificationally analysed the effect of the repair based on the recovery of the muscular morphology at the early postoperative period by the 3D-MRI reconstruction. In our research, the results demonstrated the more obvious degeneration of the incised but unrepaired muscle comparing to the repaired. In addition, as the anatomical characteristic of the short external rotators, the root of the internal obturator muscle is connected to the levator ani muscle which is closely involved in supporting the pelvic organs. Tomonori Baba et al. found that the symptoms of urinary incontinence were significantly aggravated after THA via the posterior approach due to the internal obturator muscle damage. If the tension and strength of the external rotators recovers, support of the pelvic organs and urinary incontinence may be improved [32]. Although there were no postoperative complications of prosthesis dislocation and urinary incontinence in this study, the muscle damage as well as the significant effects of the effective muscle repair was obvious. More interestingly, Tetsu Yamaguchi et al. found that the reconstruction of the short external rotators had significantly higher abduction muscle strength and external rotation muscle strength, and it could improve joint stability without limiting range of motion (ROM) [33]. Therefore, we recommend the reattachment of the detached external rotators if conditions permit. We believe that the repair provides a primary tendon insertion and probably does conduce to the early postoperative muscle recovery. It is also important to note that non-in-situ suture repair can provide initial stability for muscle recovery, but muscle degeneration will still occur slowly as time passed. Whereas, due to the muscle contracture caused by the chronic diseases, it is difficult for the external rotators to be repaired in situ. Moreover, in order to further explore the value of the repair, a extended analysis about the effects of posterior reconstruction on the important hip muscle groups has been in progress.

The main limitations of the study are listed below: 1. the limited size of cohort and follow-up time; 2. Influence on the statistical power from patients lost during the follow-up. While there was not a significant difference on the baseline data of the lost and retained and the long-term effect of the repair needs to be further studied in a larger sample clinical trial.

## Conclusion

The posterior THA caused serious damage to the external rotators and early postoperative external rotation function. An effective repair useful to the early postoperative recovery of external rotators in comparison with no repair management should be considered.

## Abbreviation

EMG: electromyography
ROM: range of motion
THA: total hip arthroplasty
